# Thoracolumbar fascia mobility and chronic low back pain: Phase 2 of a pilot and feasibility study including multimodal chiropractic care

**DOI:** 10.1186/s12998-022-00455-z

**Published:** 2022-10-21

**Authors:** Robert Vining, Stephen M. Onifer, Elissa Twist, Anna-Marie Ziegler, Lance Corber, Cynthia R. Long

**Affiliations:** 1 Palmer Center for Chiropractic Research, Palmer College of Chiropractic, 1000 Brady St, Davenport, IA USA; 2 Palmer College of Chiropractic, Information Technology, 1000 Brady St, Davenport, IA USA

**Keywords:** Fascia, Low back pain, Chiropractic, Shear strain, Ultrasound

## Abstract

**Background:**

Thoracolumbar fascia mobility observed with ultrasound imaging and calculated as shear strain is lower in persons with chronic low back pain. This pilot and feasibility trial assessed thoracolumbar shear strain in persons with chronic low back pain following spinal manipulation and over an 8-week course of multimodal chiropractic care.

**Methods:**

Adults self-reporting chronic low back pain ≥ 1 year participated between September 2019 and April 2021 in a trial using ultrasound imaging to measure thoracolumbar shear strain. Ultrasound imaging occurred 2–3 cm lateral to L2-3 while participants relaxed prone on an automated table moving the lower extremities downward 15 degrees, for 5 cycles at 0.5 Hz. Pain intensity on an 11-point numerical rating scale, disability, pain interference, and global improvement were also collected. Participants received 8-weeks of twice-weekly chiropractic care including spinal manipulation, education, exercise, self-management advice and myofascial therapies. Shear strain was computed using 2 methods. The highest shear strain from movement cycles 2, 3, or 4 was averaged over right and left sides for each participant. Alternately, the highest shear strain from movement cycle 3 was used. All data were analyzed over time using mixed-effects models. Estimated mean changes are reported.

**Results:**

Of 20 participants completing 8-weeks of chiropractic care (female n = 11), mean (SD) age was 41 years (12.6); mean BMI was 28.5 (6.2). All clinical outcomes improved at 8-weeks. Mean (95% confidence interval) pain intensity decreased 2.7 points (− 4.1 to − 1.4) for females and 2.1 points (− 3.7 to 0.4) for males. Mean Roland–Morris disability score decreased by 5 points (− 7.2 to − 2.8) for females, 2.3 points (− 4.9 to 0.2) for males. Mean PROMIS pain interference T-score decreased by 8.7 points (− 11.8 to − 5.5) for females, 5.6 points (− 9.5 to − 1.6) for males. Mean shear strain at 8-weeks increased in females 5.4% (− 9.9 to 20.8) or 15% (− 0.5 to 30.6), decreasing in males 6.0% (− 24.2 to 12.2) or 2% (− 21.0 to 16.8) depending on computational method.

**Conclusion:**

Spinal manipulation does not likely disrupt adhesions or relax paraspinal muscles enough to immediately affect shear strain. Clinical outcomes improved in both groups, however, shear strain only increased in females following 8-weeks of multimodal chiropractic care.

*Trial registration* ClinicalTrials.gov registration is NCT03916705.

**Supplementary Information:**

The online version contains supplementary material available at 10.1186/s12998-022-00455-z.

## Background

The high global prevalence of low back pain (LBP) is responsible for substantial personal and societal burden, high disability, and healthcare expenditures [1–3]. Most non-cancer and non-visceral-related acute LBP is attributed to dysfunction of neuro-musculoskeletal system tissues (e.g., nerve roots, muscles, intervertebral discs, spinal joints), which can contribute to LBP either individually or collectively [4–7]. Chronic or recurrent LBP is known to be influenced by other factors, such as central sensitization and fear-avoidance beliefs, that negatively influence prognosis, symptom severity, chronicity, and coping capacity [8–11].

The thoracolumbar fascia (TLF) is a network of connective tissues implicated in both the development and propagation of chronic LBP [12]. TLF contains layers of loose and dense connective tissue that independently move across each other to facilitate body movements, a phenomenon observable using ultrasound imaging and measured as shear strain [13]. Degenerative changes within TLF such as adhesions and fibrosis occurring within or between fascial layers is thought to reduce tissue shearing capacity, abnormally re-orient tissue loads, and activate nociceptive and aberrant proprioceptive signaling [14].

Using ultrasound imaging, Langevin et al. reported reduced TLF mobility (shear strain) and remodeling (fibrosis and disorganization), in persons with chronic LBP [15, 16]. Though it is not known if TLF changes cause, or result from, chronic LBP, TLF microinjury and/or inflammation influences nociceptor activation and body movement patterns through a series of interrelated mechanisms that also include aberrant afferent input, central nervous system nociceptive sensitization, and maladaptive tissue remodeling [12, 17, 18].

Manual therapies are thought to influence myofascial tissues such as the TLF through stretch and manual pressure, by disrupting adhesions, improving or stimulating lymphatic and vascular circulation, and reducing abnormally high muscle tone [19–21]. Hyaluronan, a glycosoaminoglycan polymer, which normally functions as a lubricant between fascial layers, is compromised with immobility, inflammation, and tissue injury [22–24]. Manual therapies temporarily alter intercellular fluid pressures [25], promoting redistributed hyaluronan within and between fascial layers [23, 26, 27], and potentially serving as a mechanical catalyst for self-resolving inflammation [28]. Manual therapies can also reduce nociception, influencing movement patterns [29].

Spinal manipulation is a guideline-recommended therapy for most non-pathological conditions causing chronic LBP, conveying a consistent beneficial therapeutic effect [30, 31]. Spinal manipulation may influence TLF mechanically by stretching or disrupting adhesions through manually generated shearing forces, and/or by facilitating reduced paraspinal muscle tone [32–35]. Other non-pharmacological interventions such as education and therapeutic alliance can potentially promote increased healthy movement by fostering self-efficacy and engaging endogenous neural pain modulation processes [36–39]. These interventions may individually or collectively help improve maladaptive TLF remodeling, thereby influencing measurable TLF shear strain and either directly or indirectly reducing pain and disability.

As an emerging field of study, a deeper understanding of the relationship between TLF shear strain and chronic LBP is needed. For example, it is unknown if TLF shear strain is altered immediately after an intervention such as spinal manipulation. It is also unknown if reduced TLF shear strain in persons with chronic LBP can be reversed/improved with a course of multimodal chiropractic care consisting of interventions directly and indirectly interacting with TLF, such as spinal manipulation, manual myofascial tissue-oriented therapies, exercise, and education.

To begin to address these questions, we conducted a pilot and feasibility study in 2 Phases to inform future randomized, controlled, and powered trials. Phase 1 enrolled participants with chronic LBP to determine the feasibility of repeatedly measuring TLF shear strain with ultrasound imaging, examining the effect of paraspinal muscle contraction, and assessing short-term stability. Phase 2 included a clinical trial to: (1) investigate the immediate effect of high-velocity, low amplitude spinal manipulation on TLF shear strain; and (2) measure potential changes in TLF shear strain over a course of multimodal chiropractic care. This article reports results obtained during Phase 2. Results from Phase 1 are reported separately.

## Methods

We conducted a single-arm pilot and feasibility trial from September 2019 to April 2021 at the Palmer Center for Chiropractic Research, Palmer College of Chiropractic, in Davenport, IA. The trial was approved by the Palmer College of Chiropractic, Institutional Review Board (B2019-006-PCCR). The ClinicalTrials.gov registration is NCT03916705.

### Recruitment

Participants in the local community learned about the trial through online advertisements, a press release, and a College webpage. Potential participants contacting the trial site by phone received a brief description of the study. Online recruitment sources linked to a College webpage included the same information. Those interested after obtaining basic information about the trial answered preliminary eligibility questions either via phone or through online questions. Eligible participants then qualified for a Baseline Visit.

### Eligibility

Eligible participants were: between 21 and 65 years old with LBP of ≥ 1-year duration and with pain on at least ½ of the days over the prior 6-months [40]; able to understand study procedures; willing to sign the informed consent document; willing to avoid any manual therapy treatment for LBP over the initial 4-week study period (Phase 1); and willing to avoid any non-trial delivered manual therapy treatment during the 8-week clinical trial (Phase 2). Exclusion criteria included: chiropractic care or other manual therapy treatment within 90 days; inability to observe necessary tissue layers using ultrasound imaging; bodyweight above 158.76 kg (350 lbs.); inability to tolerate or safely receive study procedures according to protocol; need for referral to another provider; inability or unwillingness to comply with study procedures; current or planned pregnancy (self-reported) within the study timeframe; previous thoracolumbar region surgery; need for a proxy; and connective tissue disorders such as Marfan and Ehlers-Danlos syndromes.

### Phase 1

**Fig. 1:**
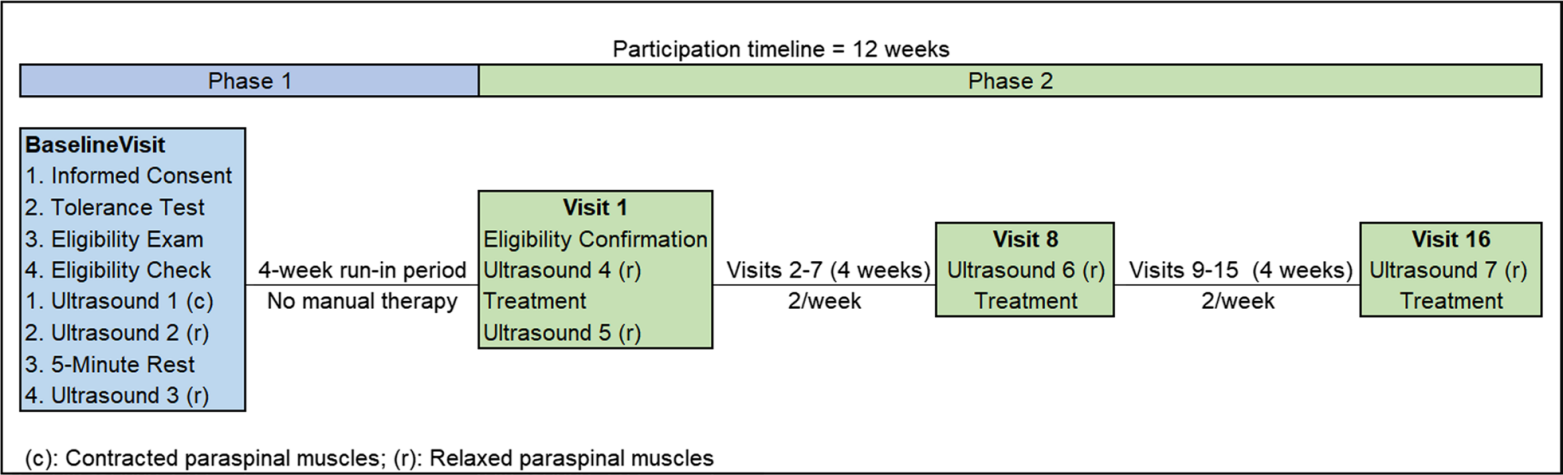
Trial flow diagram

As described in more detail elsewhere, the study began with a Baseline Visit that opened with an informed consent process (Fig. 1). Participants met with study personnel who provided a detailed description of both study phases. Following discussion with consent materials, participants reviewed images demonstrating key activities (e.g., ultrasound imaging and multimodal chiropractic care). Ample opportunity to ask questions was also provided. After obtaining written consent, demographic data and participant-reported outcome measures were collected in REDCap (Research Electronic Data Capture, Vanderbilt University, Nashville TN).

**Fig. 2:**
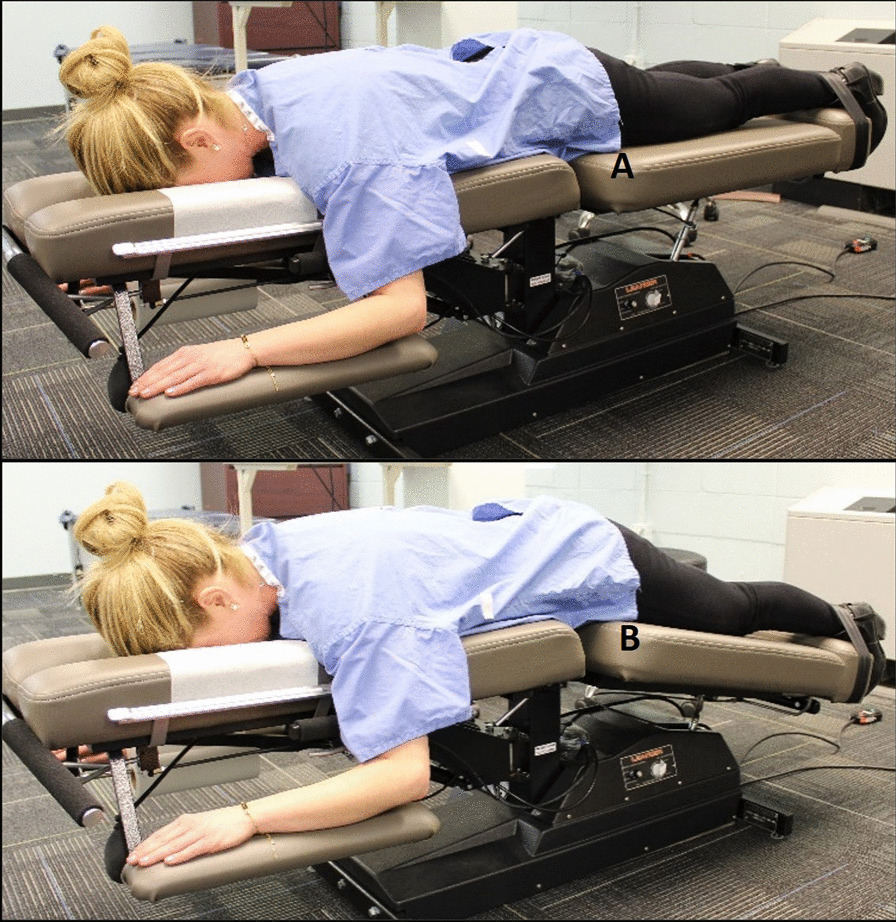
Participant positioning for ultrasound imaging. An automated table slowly moves the lower extremities from a neutral position (**A**), 15 degrees downward **B** and back to neutral, cycling 5 times over 10s

Participants then received an eligibility exam by a licensed doctor of chiropractic (DC). The evaluation comprised a health history review, a low back evaluation [41], and a brief ultrasound imaging evaluation to determine if key TLF tissue layers were visible. Tolerance to ultrasound imaging procedures was assessed while lying prone and relaxed on an automated motorized table (Leander LT 950, Lawrence, KS) with a moveable section supporting the lower extremities. The anterior superior iliac spine was located bilaterally at the cephalic end of the moveable support. The table moved the lower extremities 15 degrees downward for 5 cycles over 10 s (Fig. 2). Discomfort, pain, or other sensations preventing conscious relaxation rendered participants ineligible because of the potential to cause conscious or unconscious trunk muscle guarding. Discomfort suggesting the ultrasound imaging procedure might be harmful (e.g., peripheralizing symptoms) was also exclusionary.

As shown in Fig. 1, three separate sets of ultrasound imaging (obtained bilaterally and referred to as Ultrasound 1, 2, and 3) followed eligibility confirmation at the Baseline Visit. Afterward, participants entered a 4-week no manual therapy run-in period. Ultrasound 4 was obtained on Visit 1. Ultrasound 4 provided the final shear strain data for Phase 1 and baseline shear strain data for Phase 2.

### Ultrasound imaging

We used a Terason T3000 ultrasound system (Teratech Corporation, Burlington, MA) with a Terason 12L5 transducer set at 10 MHz and programmed to record a cine-loop for 20 s in B-mode at a 25 Hz frame rate. Ultrasound imaging occurred individually for each side with participants prone on the automated table moving the lower extremities and cycling 5 times at 0.5 Hz (2 s per cycle). The ultrasound transducer was placed parallel to the spine, approximately 2–3 cm lateral to the L2-3 spinous process interspace. Ultrasound recordings were conducted according to pre-defined protocols. To ensure consistent data collection, each of 3 study personnel were assigned specific roles (ultrasound transducer operator, ultrasound unit operator, participant guide). The ultrasound transducer operator confirmed fascial layer visualization before each recording. Once visualized, the transducer was stabilized manually without tissue compression and with surgical tape on the cephalic side to reduce transducer movement artifact. Each recording began 2–4 s before initiating table movement, ending 2–4 s after table movement ceased. Completed recordings then underwent a quality control process to ensure fascial layers were visible during the entire recording and the transducer remained stable. If either condition was unmet, the recording was repeated.

After recording, ultrasound imaging files were converted to a file format compatible with a custom MATLAB (The MathWorks, Inc, Natick, MA) data processing program. Files were also renamed with a 5-digit randomly generated number and transferred to a consultant for processing. File transfers used Microsoft OneDrive, a secure encrypted cloud storage sharing service.

**Fig. 3:**
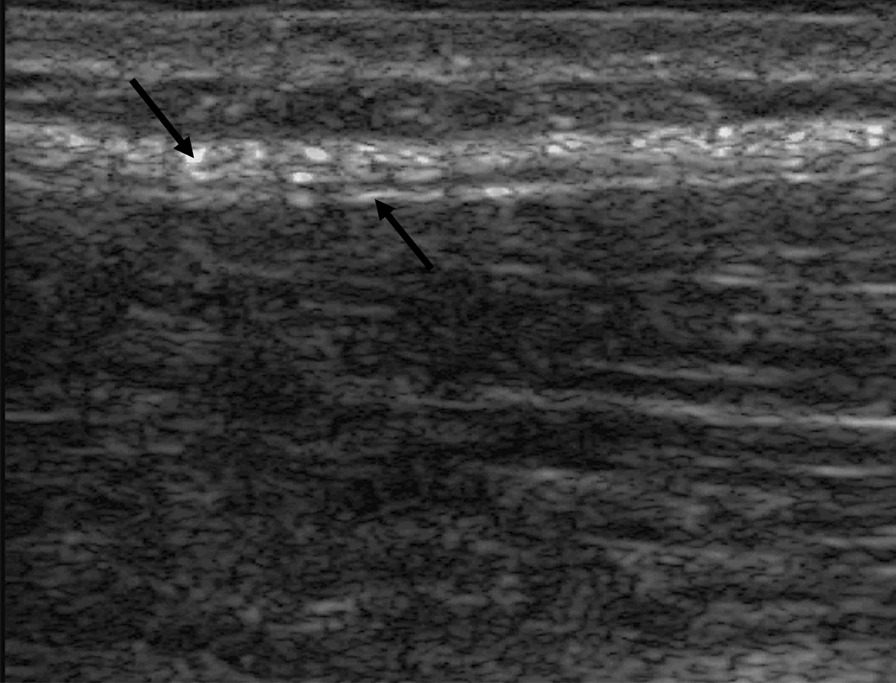
Parasagittal ultrasound image of thoracolumbar fascia at the L2-3 level. Left downward arrow: Thick echogenic layer representing the aponeurosis of the latissimus dorsi and abdominal muscles. Right upward arrow: Thin echogenic layer representing the aponeurosis surrounding paraspinal muscles. Between the arrows is a thin echolucent layer of loose connective tissue. Deep to the thin echogenic layer are paraspinal muscles. Shear strain represents differential motion (moving laterally on the image) between echogenic layers

Converted ultrasound imaging files were processed by a consultant who developed and reported processing methods in detail [15, 16, 32] using a custom program written in MATLAB. Shear strain calculation involved measuring differential motion between an echogenic layer representing the aponeurosis of the paraspinal muscles and another echogenic layer representing the aponeurosis of the latissimus dorsi and abdominal wall muscles (Fig. 3). These two layers are separated and distinguished by a thin echolucent layer of loose connective tissue that lies between them. Tissue displacement (motion) was estimated using cross-correlation techniques. Tissue axial and lateral displacement was computed from radiofrequency data acquired at each time point in a 1X1.5 cm region of interest (ROI).

To allow for uncertainty of the true location of the echolucent layer from confounding axial motion, sub-ROI’s were positioned initially at − 0.5 mm, 0.0 mm, and + 0.5 mm relative to the initial position. Shear strain was calculated as the largest absolute differential motion between superficial and deep fascial layers observed during cycles 2, 3, and 4, divided by the distance (1 mm) between the centers of the 2 sub-ROIs, and expressed as a percentage. Automated tracking accounted for ROI vertical movement on the image. When detected, ROI location was adjusted. Additional details describing data processing methods are reported by Langevin et al. [16].

### Phase 2

As a pilot study, we planned to assess the feasibility of an 8-week protocol of bi-weekly multimodal chiropractic care including 3 visits with ultrasound imaging (Fig. 1). Recruitment data from prior studies using a wait-list period at our center suggested approximately 40 participants would be enrolled in Phase 1 to achieve the intended goal of 30 participants (15 female and 15 male) in Phase 2.

During the study, all participant-related activities were interrupted for a 5-month period due to the COVID-19 pandemic. Participants active at trial interruption were contacted when study activities resumed. Those still interested in participating and able to attend visits were scheduled for the next visit in sequence, as if the trial was not interrupted.

Phase 2 eligibility was assessed with a case review process, with final eligibility confirmation on Visit 1 (Fig. 1) [42]. During Visit 1, study DCs provided a report of findings from the Baseline Visit exam and inquired about recent changes in health status. A Study Coordinator then reviewed study information to ensure ongoing understanding of the clinical trial. The Study Coordinator and study DC then jointly verified Phase 2 eligibility.

Ultrasound 4 was obtained during Visit 1 immediately after completing patient-reported outcomes. To assess immediate effects of spinal manipulation on TLF shear strain, Ultrasound 5 was obtained immediately after participants received manually delivered spinal manipulation to the lumbopelvic and/or thoracic regions. Fifteen more visits including multimodal chiropractic care occurred over 8 weeks. Ultrasound imaging also occurred at the beginning of Visit 8 (Ultrasound 6) and Visit 16 (Ultrasound 7) (Fig. 1).

### Patient-reported outcome measures

We collected patient-reported outcomes at the beginning of Visits 1, 8, and 16: (1) average pain intensity over the past 7 days using an 11-point numerical rating scale [40, 43]; (2) disability using the Roland–Morris Disability Questionnaire (RMDQ) [44]; and (3) the PROMIS 4-item Pain Interference instrument [40]. Perceived Global LBP Improvement was assessed at the beginning of Visit 16 using a single question: “Compared to your first visit, your low back pain is:” Responses included: Completely gone (0), Much better (1), Moderately better (2), A little better (3), About the same (4), A little worse (5), or Much worse (6).

### Multimodal chiropractic care

On Visit 1, only manually delivered spinal manipulation was used between Ultrasound 4 and 5 to ascertain immediate effects on TLF shear strain. Personalized home exercise recommendations, education, and advice were offered at the end of the visit after Ultrasound 5. Bi-weekly chiropractic care on subsequent visits included a multimodal approach consisting of 4 often co-occurring components [45]: (1) education: to provide information about working diagnoses, including chronic pain, with the goal of enabling participants to better understand and interpret symptoms, to promote self-efficacy, and improve health literacy; (2) passive interventions: controlled and performed by a clinician such as spinal manipulation and myofascial therapies; (3) active interventions: Exercise and mindfulness-based interventions controlled and performed by participants to enhance treatment effectiveness, build strength, endurance, muscular coordination, self-efficacy, and/or reduce kinesiophobia and pain sensitivity; and (4) self-management advice/activities designed to help individuals self-monitor, control and/or reduce the impact of LBP over time. Additional file 1 describes multimodal chiropractic care in this trial using a template for intervention description and replication checklist format [46].

### Adverse events

Questions probing for potential adverse events were asked at each visit. Adverse events were defined as any untoward medical occurrence identified during trial participation, regardless of causal relationship with research-related procedures [47, 48]. All identified events were graded by study clinicians on 3 levels: severity (mild, moderate, severe, serious), expectedness (unexpected, expected), and relatedness (unrelated, unlikely, possibly, probably, definitely) to participating in the trial. All adverse events were reviewed by a separate clinician for clarity and to verify grading. Discrepancies were resolved through consensus discussion.

### Blinding

Study DCs were blind to patient-reported outcomes and shear strain data throughout the trial. The consultant was blind to all ultrasound imaging conditions (Ultrasound 4, 5, etc.) and potential identifying information (e.g., sex) through the randomly generated number file renaming process.

### Analysis

Mean maximum shear strain for each ultrasound was calculated by averaging the maximum shear strain observed on each side during a single table motion cycle (cycle #3), consistent with methods reported by Langevin et al. [16]. Because maximum shear strain may not always occur on cycle #3, we also calculated shear strain by choosing the maximum shear strain observed on cycles 2, 3, or 4 on each side, prior to averaging. Participants completing the 8-week intervention protocol were included in the analyses. All data was used in this longitudinal analysis except for participants whose care was interrupted by the COVID-19 pandemic suspension period; only data up until the study suspension was used for these participants.

SAS (release 9.4; SAS Institute, Inc., Cary, NC) was used to analyze data. The trial was not powered. Analyses were conducted for each of the 2 shear strain measures and assumptions were accessed with residual analyses. Shear strain measures were included in mixed-effects regression models using individual participants as a random effects to account for repeated measures and an unstructured covariance. Fixed effects were time, sex and a time x sex interaction in order to report effect sizes with 95% confidence intervals for each sex, because TLF shear strain has been shown to vary by sex [16]. We used contrasts to obtain changes over time. The mixed-effects models were used to analyze shear strain pre- and post-spinal manipulation at Visit 1 and over pre-spinal manipulation at Visits 1, 8, and 16. The same mixed effects models were used to analyze patient-reported outcome variables over visits 1, 8 and 16,

## Results

**Fig. 4:**
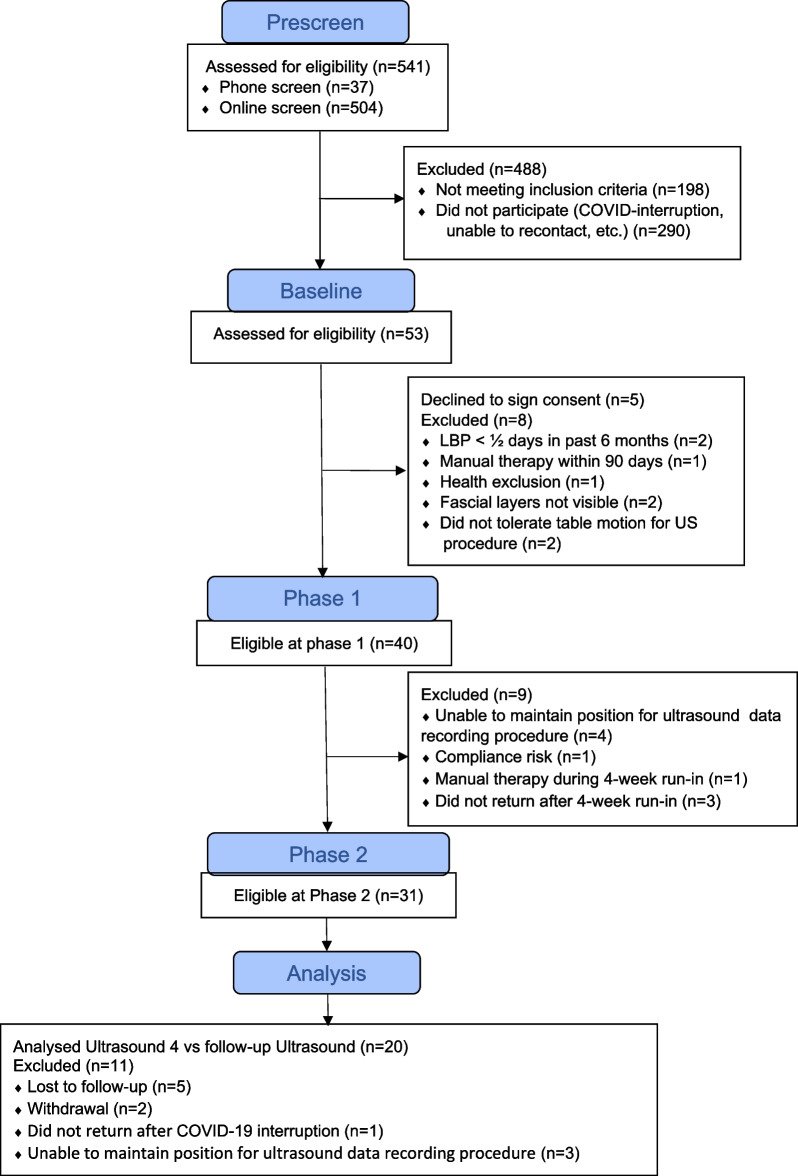
CONSORT 2010 flow diagram

The study was conducted between September 2019 and April 2021, including a 5-month interruption pausing all participant-related activities due to the COVID-19 pandemic. Five-hundred forty-one participants were screened either with an online questionnaire or via phone (Fig. 4). Of these, 53 attended Baseline Visits with 40 participants included in Phase 1, and 31 in Phase 2. Participants active when the study was interrupted, whether in Phase 1 or Phase 2, were rescheduled to complete the next scheduled visit if still interested and able to attend appointments when the study resumed.

Of the 31 participants eligible for Phase 2, 3 were identified mid-trial as unable to rest the head fully on the table during ultrasound imaging. This created potential involuntary paraspinal muscle contraction and an opposing stretching force while the head acted as a counterweight during ultrasound imaging. One participant did not return from the COVID-19-related interruption, and 7 did not complete the 8-week trial. Participant-reported outcome measures and ultrasound imaging data from the remaining 20 were used in the analyses.

Table 1 displays demographic characteristics of Phase 2 participants. Because prior research suggests potential differences in TLF function between males and females, outcomes are reported by sex [16]. At baseline, females had higher pain intensity, disability, and pain interference than males.

**Table 1:** Participant demographic characteristics (n = 20)

	Females (n = 11)	Males (n = 9)	Total
Age, mean (SD, range)	42 (13.2, 22–61)	40 (12.5, 23–61)	41 (12.6, 22–61)
BMI, mean (SD)	27.1 (6.5)	30.1 (5.7)	28.5 (6.2)
Ethnicity, n (%)			
Hispanic or Latino	0 (0)	1 (5)	1 (5)
Race, n (%)			
American Indian/Alaska Native, Asian, and Black or African American	0 (0)	1 (5)	1 (5)
White	8 (40)	7 (35)	15 (75)
Multi-race	3 (15)	1 (5)	4 (20)
Highest education level, n (%)			
High school	2 (10)	1(5)	3 (15)
College	8 (40)	8 (40)	16 (80)
Graduate or professional degree	1 (5)	0 (0)	1 (5)
Employment status, n (%)			
Full-time	8 40)	4 (20)	12 (60)
Part-time	0 (0)	3 (15)	3 (15)
Unemployed	2 (10)	1 (5)	3 (15)
Unspecified	1 (5)	2 (10)	1 (5)
Average pain, mean (SD)	5.5 (1.6)	4.6 (2.4)	5.1 (1.97)
Roland-Morris disability questionnaire score, mean (SD) (0–24, 0 = no disability, 24 = severe disability)	8.2 (4.2)	5.3 (3.6)	6.9 (4.14)
PROMIS^®^ (T score), pain intensity, mean (SD)	62.4 (4.8)	55.8 (7.7)	59.5 (6.92)

Table 2 displays mean clinical outcomes at Visits 1, 8, and 16. Mean (95% Confidence Interval) pain intensity reduction over 8-weeks was 2.7 (− 4.1 to − 1.4) for females and 2.1 (− 3.7 to 0.4) for males on an 11-point numerical rating scale. Mean Roland–Morris Disability Questionnaire scores decreased by 5 points (− 7.2 to − 2.8) for females and 2.3 points (− 4.9 to 0.2) for males. Mean PROMIS Pain Interference T-scores decreased by 8.7 points (− 11.8 to − 5.5) for females and 5.6 points (− 9.5 to − 1.6) for males. Median Perceived Global Improvement at visit 16 was 2.0 (moderately better) (interquartile range: 2 to 3) for both females and males.

**Table 2:** Mean clinical outcomes over 8 weeks (n = 20) (−: decreased)

	Visit 1 mean (SE)	Visit 8 mean (SE)	Change at visit 8 (from baseline) mean (CI)	Visit 16 mean (SE)	Change at visit 16 (from baseline) mean (CI)
*Females (n = 11)*					
Average pain (NRS)	5.5 (0.6)	4.1 (0.6)	− 1.3 (− 2.6 to 1.1)	2.7 (0.6)	− 2.7 (− 4.1 to − 1.4)
Roland-Morris disability questionnaire	8.2 (1.2)	6.7 (1.4)	− 1.5 (− 3.3 to 0.4)	3.2 (0.7)	− 5.0 (− 7.2 to − 2.8)
PROMIS: pain interference	62.4 (1.9)	58.1 (2.0)	− 4.4 (− 8.2 to − 0.5)	53.8 (2.1)	− 8.7 (− 11.8 to − 5.5)
*Males (n = 9)*					
Average Pain (NRS)	4.7 (0.7)	2.8 (0.6)	− 1.9 (− 3.4 to 0.4)	2.5 (0.7)	− 2.1 (− 3.7 to 0.4)
Roland-Morris disability questionnaire	5.3 (1.3)	3.7 (1.5)	− 1.6 (− 3.7 to 0.5)	3.0 (0.9)	− 2.3 (− 4.9 to 0.2)
PROMIS: pain interference	55.8 (2.1)	50.6 (2.3)	− 5.2 (− 9.5 to − 0.9)	50.3 (2.4)	− 5.6 (− 9.5 to − 1.6)

*SE* standard error; *CI* 95% confidence interval

Table 3 displays mean shear strain pre- and immediately post spinal manipulation using 2 computational methods: (1) mean maximum shear strain during the 3rd movement cycle; and (2) mean maximum shear strain measured on cycle 2, 3, or 4. Mean shear strain in females immediately after spinal manipulation increased 2.1% (− 10.5 to 14.6) using method 1 and decreased 4.5% (− 19.2 to 10.3) using method 2. In males, mean shear strain decreased 0.3% (− 14.2 to 13.6) using method 1 and increased 5.8% (− 10.5 to 22.1) with method 2.

**Table 3:** Mean % Shear Strain pre- and immediately post-spinal manipulation (n = 20) (-: decreased)

	Females (n = 11)			Males (n = 9)		
	Pre spinal manipulation mean (SE)	Post spinal manipulation mean (SE)	Change post spinal manipulation Mean (CI)	Pre spinal manipulation mean (SE)	Post spinal manipulation mean (SE)	Change post spinal manipulation mean (CI)
Shear strain (calculation method 1)	56.8 (7.0)	58.9 (6.9)	2.1 (− 10.5 to 14.6)	55.2 (7.7)	54.9 (7.6)	− 0.3 (− 14.2 to 13.6)
Shear strain (calculation method 2)	76.7 (7.1)	70.2 (7.2)	− 4.5 (− 19.2 to 10.3)	68.3 (7.8)	74.1 (8.0)	5.8 (− 10.5 to 22.1)

*SE* standard error; *CI* 95% confidence interval; Calculation method 1: mean maximum shear strain, from the left and right sides, observed during the 3rd table movement cycle; Calculation method 2: mean maximum shear strain, from the left and right sides observed during any table movement (cycles 2, 3, or 4)

Table 4 displays mean shear strain at Visits 1, 8, and 16. At Visit 16, mean shear strain in males decreased by 2.1% (− 21.0 to 16.8) using method 1, and 6.0% (− 24.2 to 12.2) using method 2. For females, mean shear strain increased with both computational methods, 15% (− 0.5 to 30.6) using method 1 and 5.4% (− 9.9 to 20.8) using method 2.

**Table 4:** Mean % Shear Strain over 8-week course of multimodal chiropractic care (n = 20)

	Visit 1 pre mean (SE)	Visit 8 pre mean (SE)	Change at visit 8 (from visit 1) mean (CI)	Visit 16 pre mean (SE)	Change at visit 16 (from visit 1) mean (CI)
*Females (n = 11)*					
Shear strain (calculation method 1)	56.8 (7.0)	55.4 (6.0)	− 1.5 (− 20.3 to 17.4)	71.9 (7.1)	15 (− 0.5 to 30.6)
Shear strain (calculation method 2)	76.7 (7.1)	69.6 (6.9)	− 6.0 (− 23.3 to 11.2)	80.1 (6.0)	5.4 (− 9.9 to 20.8)
*Males (n = 9)*					
Shear strain (calculation method 1)	55.2 (7.7)	48.5 (6.7)	− 6.7 (− 27.6 to 14.2)	53.1 (8.7)	− 2.1 (− 21.0 to 16.8)
Shear strain (calculation method 2)	68.3 (7.8)	64.5 (7.8)	− 3.8 (− 23.0 to 15.4)	62.3 (7.4)	− 6.0 (− 24.2 to 12.2)

*SE* standard error; *CI* 95% confidence interval; Calculation method 1: mean maximum shear strain, from the left and right sides, observed during the 3rd table movement cycle; Calculation method 2: mean maximum shear strain, from the left and right sides observed during any table movement (cycles 2, 3, or 4)

### Adverse events

Forty-seven mild and 4 moderate adverse events among all 31 participants during Phase 2 were graded as either possibly (n = 16), probably (n = 21), or definitely (n = 14) related to study participation. Thirty-seven mild events were attributed to treatment, 8 to ultrasound imaging, and 2 to a combination of study procedures. Most mild events included temporary increases in LBP, stiffness, muscle and joint soreness, and occasional radiating pain lasting from minutes to hours (n = 37) to a few days (n = 8). Two participants experienced mild symptom increases for approximately 2 weeks.

Four moderate events among 3 participants were graded as possibly (n = 1) or probably (n = 3) related to treatment. One participant reporting a recurrence of lower extremity pain and numbness beginning a few days after a study visit sought care from a non-study provider. Another participant reported increased symptoms prompting rest and reduced physical activities on 2 separate occasions. A third participant reported increased LBP and radiating symptoms lasting approximately 3 days, prompting self-directed symptomatic (heating pad) therapy. Mild events attributed to ultrasound imaging procedures (n = 8) included temporary increases in low back pain, uncomfortable stretching, and/or burning sensations.

## Discussion

Results from this study are consistent with those of Langevin et al. [16], who first reported sex-based differences in TLF shear strain (63% females and 51% males with chronic LBP) using the same data collection procedure while measuring shear strain data obtained from the 3rd table movement cycle. In our study, using the same data collection procedure and considering shear strain only on the 3rd table movement cycle, mean shear strain in females observed at Visit 1 was 57% and 55% in males.

However, using shear strain only from the 3rd table movement cycle presumes the highest shear strain occurs only on cycle 3. It is conceivable that higher shear strain can be observed on other table movement cycles as was the case in this trial. Therefore, we also computed shear strain using the highest observed from any of the middle table movement cycles (2, 3, or 4). When using the highest measured shear strain on any of cycles 2, 3, or 4, theoretically capturing the maximum differential motion between fascial layers, the difference between females (77%) and males (68%) was consistent with the 12% difference noted by Langevin et al. [16].

Changes in shear strain immediately following spinal manipulation differed depending on computational method. Regardless of method, changes were minimal, suggesting that if spinal manipulation consistently influences shear strain in people with chronic LBP in the short term, due either to disrupting adhesions between fascial layers or from muscle relaxation, the effect is very small. Marked and sustainable changes in TLF shear strain in people with chronic LBP likely require biochemical and anatomical adaptations that occur over longer time periods [29, 49].

Pain intensity, disability, and pain interference improved in both males and females after 8-weeks, consistent with previous studies including multimodal chiropractic care for chronic LBP [50, 51]. Mean shear strain also improved in females. However, a similar improvement in males was not observed. These differential results could suggest an association between shear strain and clinical improvement in females whereas the relationship either does not exist or is distinctly different in males. Langevin et al., reported a moderate relationship between thoracolumbar connective tissue thickness and echogenicity (evidence of fascial remodeling) with reduced range of motion and physical function in males with chronic LBP. These findings suggested these relationships may be linked in males because of unique factors such as body composition, fat distribution, hormonal influences, and/or movement patterns. Findings from this study similarly suggest different mechanisms influence thoracolumbar fascia in males and females.

Should these results be confirmed, new questions related to ultrasound imaging and shear strain arise such as: what mechanisms explain sex-based differences in TLF shear strain? Does shear strain similarly improve in males at a slower rate? Is there a threshold of clinical outcome changes needed before associated improvements in TLF shear strain occurs? What is the appropriate timeframe to assess shear strain for patients receiving care for chronic LBP? Can shear strain be used as an objective indicator of functional improvement? How do anatomically visible changes in TLF such as fibrosis and perimuscular thickness relate to TLF shear strain and clinical outcomes in persons with chronic LBP [52, 53]?

### Limitations

This feasibility/pilot trial was not powered and therefore is limited by a small sample size leading to wide confidence intervals observed in trial results. The trial was also limited by an abrupt 5-month interruption of study activities due to the COVID-19 pandemic. The ultrasound imaging method was also a limitation. As observed in Phase 1 of this study, paraspinal muscle contraction temporarily reduced measured shear strain. Therefore, voluntary or involuntary paraspinal muscle guarding during ultrasound imaging may have influenced shear strain results. Subsequent research using these methods may be further informed by simultaneously collecting ultrasound and paraspinal muscle contraction data. Limitations also include the absence of a control group. Because of the small sample size and lack of control group, connective tissue thickness and echogenicity were not measured to assess possible relationships with clinical outcomes or differences in shear strain over time. Despite these limitations, this trial: (1) demonstrated the feasibility of collecting sequential shear strain measurements during a course of multimodal chiropractic care; (2) corroborated evidence for differences in shear strain between males and females; (3) demonstrated sex-based differences in TLF shear strain after a course of care for chronic LBP; and (4) identified additional research questions.

## Conclusion

Clinical outcomes improved in males and females over an 8-week course of multimodal chiropractic care consisting of education, exercise, spinal manipulation, myofascial therapies, and self-management advice. Shear strain increased in females over the same timeframe. However, shear strain did not similarly increase in males. Immediate shear strain changes following spinal manipulation applied to the thoracic or lumbo-pelvic areas suggested effects were either small or absent.

Further research is needed to determine functionally meaningful changes in shear strain. Research including control groups in sufficiently powered trials can enable a deeper understanding of shear strain changes over longer timeframes and the relative contribution of individual interventions to those changes. Future research focused on understanding mechanisms responsible for sex-based differences in TLF shear strain may also help inform more personalized care for persons with chronic LBP.

## Supplementary Information

**Additional file 1**. Intervention Description.

## Data Availability

Datasets used for this study may be available through request to the corresponding author on reasonable request.

## Abbreviations

LBP: Low back pain
TLF: Thoracolumbar fascia
BMI: Body mass index
RMDQ: Roland–Morris disability questionnaire
