# Supplementary material for: Thoracolumbar fascia mobility and chronic low back pain: Phase 2 of a pilot and feasibility study including multimodal chiropractic care
Source: Chiropr Man Therap. 2022 Oct 21;30:46. doi: 10.1186/s12998-022-00455-z (PMC9587561; doi:10.1186/s12998-022-00455-z)
Supplement: Supplementary file 1 — Additional file 1. Intervention Description. [file 12998_2022_455_MOESM1_ESM.docx]

| **Name** | Multimodal Chiropractic care |
| --- | --- |
| **Why** | Multimodal chiropractic care comprises interventions that potentially influence thoracolumbar fascia (TLF). Exercise and manual therapies such as soft-tissue mobilization and stretching, impart direct mechanical effects. Indirect effects from interventions such as education and self-management advice/activities may help reduce nociception, alter pain beliefs, and pain-associated behaviors. The combined effects of manual therapies, education, and exercise may lead to changes in body movements and muscle contraction patterns, which in turn facilitate gradual tissue remodeling and improved (TLF) mobility. Including a multimodal approach in this trial facilitates answering the research question of whether reduced TLF mobility improves over an 8-week course of multimodal care. |
| **What** | Treatment tables: 1 custom height adjustable (19 in. – 31 in.) padded exam and treatment table and Eurotech chiropractic treatment tables (Osage Beach, MO)  High velocity, low amplitude spinal manipulation (HVLA-SM): HVLA-SM to the thoracic or lumbopelvic region involved specific patient positioning guided by the clinician. The manipulative load was applied with the clinician’s hand as it contacted specific areas over the participant’s thoracic or lumbar spine, or sacroiliac joint(s). The manipulative thrust was delivered by quick, short, and controlled movements arm and hand. HVLA-SM occurred with the participant in a prone, side-lying, or supine position based on individual characteristics such as comfort and tolerance. The high velocity thrust typically results in what is commonly called joint cavitation/tribonucleation. HVLA-SM was sometimes applied with the aid of a hand-held instrument (Activator Methods, Phoenix AZ), which delivers a high-velocity mechanical impulse instead of a manual thrust.  Myofascial therapy: Trunk and lower extremity muscle stretching was recommended when muscles or tissues in these regions were deemed abnormally hypertonic and/or contributing to symptoms. Ischemic compression and deep manual pressure with shearing motion was also used in the thoracolumbar or sacroiliac regions individually and per participant tolerance.  Exercises: Exercises were routinely prescribed. Exercises included: abdominal breathing, repeated range of motion movements including directional preference exercises, pelvic tilting, squatting, gluteal bridges (single and bilateral support), side plank, bird dog, and dead bug. Participants were regularly encouraged to engage in general exercise such as walking in addition to prescribed exercises. Stability-oriented exercises were recommended individually based on ability to perform. Exercises were generally prescribed in a graded manner, beginning with simple, moving toward more complex movements and positions and incorporating breathing and more challenging stability elements as capacity improved and as tolerated.  Education: Participants were informed about the working diagnosis and how this contributed to symptoms. Education regarding chronic pain sensitization and how treatment, specific and general exercises, and other lifestyle recommendations were designed to positively influence pain sensitization and contribute to improved pain and function.  Prohibited interventions: Treatment applied to the cervical (neck) region, for extremity conditions, neck pain, or headache, electrical modalities, heat/ice, and a spinal manipulative technique referred to as flexion-distraction. Though clinical guidelines recommend heat/ice as an effective treatment for LBP, their use in this study is limited to home use through self-management advice. Heat/ice is prohibited during a study visit to eliminate potential short-term changes in muscle contraction and tissue fluid dynamics that could artificially influence tissue mobility measures. Because a flexion-distraction table and motion was used during ultrasound recordings, repeated tissue stretching effects from a treatment utilizing the same procedures could potentially confound results interpretation. Therefore, flexion-distraction treatment techniques were not used in the trial. |
| **Who Provided** | Care was provided by 3 licensed doctors of chiropractic with experience ranging from 5 to 30 years. |
| **How** | All care was provided individually in a face-to-face format. |
| **Where** | All visits took place in a dedicated 2700 square foot research clinic that includes consultation, exam, treatment rooms, and a laboratory accommodating ultrasound data collection. |
| **When and How Much** | Phase 2 (clinical trial) included 16 visits over 8 weeks. Most visits were scheduled twice per week. Interventions employed at each visit varied depending on symptoms, tolerance, and response to care. |
| **Tailoring** | Consistent with the primary research question, this trial sought to emulate a pragmatic approach to chiropractic care, which is typically multimodal and individually tailored. Most visits employed some form of HVLA-SM. Other commonly employed interventions included education, exercise, myofascial treatment, and advice. |
| **Modifications** | Treatment was tailored to individuals at each visit. No changes in allowed or prohibited treatments occurred during the trial. |
| **How well** | At each visit, all treatment was recorded by category in the participant’s clinical record. Categories included: Education about condition, spinal manipulation, myofascial therapy, in-office exercise, at home exercise (prescribed), and self-management advice. All treatment records were reviewed by study coordinators following each visit. No fidelity concerns were identified. |
